# Unqualified Assistants

**Published:** 1898-12-10

**Authors:** 


### The Hospital

A Journal of

The Medical Sciences and Hospital Administration.

Vol. XXV.—No. 637.] Edited by Sir Henry Burdett, K.C.B., and Solomon C. Smith, M.D., M.R.C.P. [Dec. 10, 1898.

### UNQUALIFIED ASSISTANTS.

Perhaps the most important business transacted by the Medical Council, in the course of the session which has just been brought to a termination, was the consideration of a letter addressed by Mr. F. A. Wild, until lately an unqualified assistant to a general practitioner, to the Lord President of the Privy Council, and forwarded by his Grace; together with a communication on the same subject from the Gateshead Medical Association. Mr. Wild complained that he had been dismissed from his situation, and the members of the Gateshead Medical Association complained that they did not know how to act, in consequence of the issue by the Council of a notice setting forth that the employment of an unqualified assistant to *attend or treat patients in matters requiring professional knowledge and skill* would be regarded by the Council as "in its nature fraudulent and dangerous to the public health," with the inevitable consequence that a practitioner who was proved to have pursued such a course would be liable to be adjudged guilty of "infamous conduct in a professional respect, and to have his name erased from the Medical Register." It was added that the notice did not apply to the proper training and instruction of *bonâ fide* medical students as pupils, or the legitimate employment of dressers, midwives, dispensers, and surgery attendants, under the immediate personal supervision of registered medical practitioners." Mr. Wild's complaint merely expressed his sense of personal hardship at being deprived of his occupation, and his desire to be reinstated; but the members of the Association declared their inability to interpret the terms of the notice, and put a series of categorical questions, such, for example, as: "Is *any* man who has registered as a student, but has not yet passed his final examination, a *bonâ fide* student? or, if not, how many years must elapse after registration before he ceases to be so?" There were several other inquiries of a similar kind, and the querists, who entirely disclaimed "a carping spirit," said that their desire was to carry out the wishes of the Council, but that they entertain widely differing ideas of the meaning which the words used are intended to convey.

The most important observation of the committee to which the several communications mentioned were referred is that they make it plain "that the evils against which the recent action of the Council was directed were more widespread and deeply rooted than was probably realised either by the profession or the public"; and that, although the removal of these evils must produce a certain amount of temporary difficulty or even hardship, yet that the paramount duty of the Council in regard to the public renders it necessary that the law should be strictly enforced. The fact is—and it is one which has been brought very much under of the Council in the course of its in the notice vestigations of charges of "covering" unqualified practitioners—that a very large number of medical men in certain districts have for many years been in the habit of using unqualified assistants as if they were qualified—that is to say, of employing them to visit and attend patients on their own responsibility, and practically without any supervision of their conduct. These unqualified assistants were mostly derived from students of the class known as "chronics" at all medical schools; and it was often said of them that they were "students" who had been prevented by "circumstances" from obtaining a qualification. In many instances, however, the so-called "practice" really belonged to the assistant, who was, in fact, the employer of his supposed master; while in others a single qualified man, by keeping two or even three unqualified assistants, was able to cover a much larger area of practice than would otherwise have been possible, to exclude qualified men, and to undersell them if they came into the neighbourhood of his preserve. The results to patients were constantly disastrous, while it is manifest that the profession, by employing quacks itself, greatly weakened its own power of protesting against quackery. The Council, on the report of its committee, has absolutely declined to issue the formal definitions asked for, and points out that the terms are used in their ordinary and natural sense. The "students" sanctioned are such as are honestly pursuing their medical studies with a view to obtaining a qualification in regular course. By the "immediate personal supervision" of such students as pupils it is meant that the practitioner must ensure that they are properly taught, and also that the welfare of his patients is in no way prejudiced by their acts. Each particular case which comes before the Council must be dealt with upon its merits, and with due consideration of all the evidence adduced.

